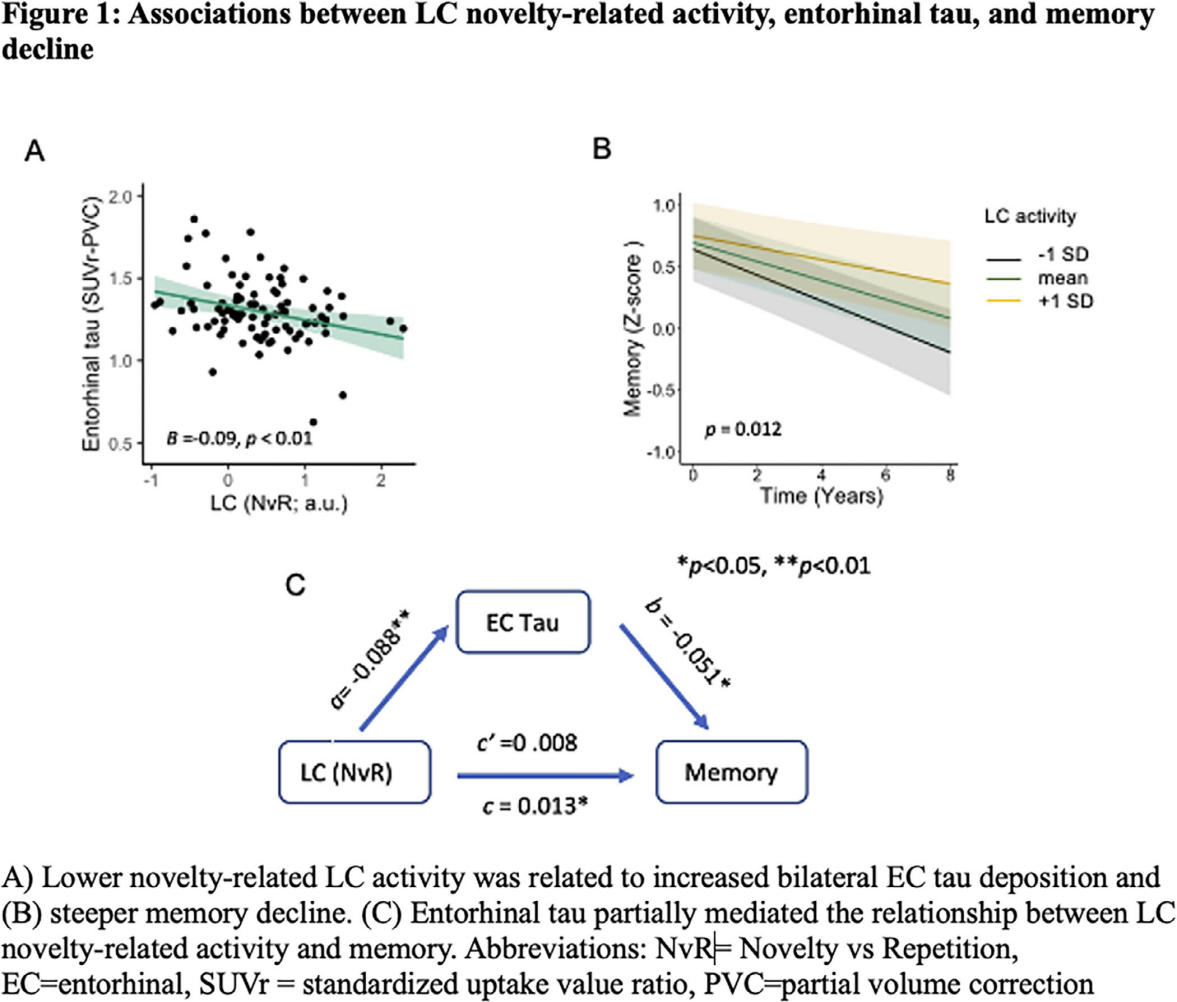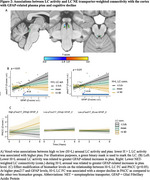# Specific functional patterns of the locus coeruleus and cognitive decline in preclinical Alzheimer's disease: associations with tau and astrocyte reactivity

**DOI:** 10.1002/alz70856_100816

**Published:** 2025-12-25

**Authors:** Heidi I.L. Jacobs, Prokopis C. Prokopiou

**Affiliations:** ^1^ Athinoula A. Martinos Center for Biomedical Imaging, Department of Radiology, Massachusetts General Hospital, Harvard Medical School, Charlestown, MA, USA

## Abstract

**Background:**

Autopsy studies suggest that the locus coeruleus‐ norepinephrine (LC‐NE) system is one of the first regions to accumulate tau in Alzheimer's disease (AD). Previous CSF studies reported that tau pathology covaried with higher NE metabolic turnover and conjointly contributed to disease progression in early AD. We examined whether increased LC activity is also linked to tau and cognitive decline in the preclinical stages of AD using specific task fMRI paradigms expected to elicit higher bursts of LC neuronal firing.

**Method:**

Two cohorts were examined: 1) 92 asymptomatic older individuals from the Harvard Aging Brain Study (mean:69.62 years, 55% female, CDR=0) who underwent task fMRI (novelty), Aβ and tau‐PET and longitudinal cognitive testing up to 8 years. 2) 79 individuals (mean:61 years, 53% female, CDR=0) who underwent 7T fMRI (arousal task), 7T LC‐imaging, longitudinal cognitive testing up to 4 years, and phlebotomy for plasma biomarkers (GFAP, ptau217, NfL). Linear regression and mixed‐effects analyses associated task fMRI contrasts (novelty vs repetition | high vs low arousal) with tau and cognitive decline, with age, sex and education as covariates.

**Result:**

1) Lower novelty‐related LC activity was associated with greater entorhinal/medial temporal lobe tau and steeper memory decline. The relationship between LC activity and memory decline was mediated by entorhinal tau, particularly in individuals with elevated beta‐amyloid (Figure 1). 2) Lower high arousal LC activity and NE transporter‐weighted connectivity with the cortex were associated with higher ptau217, particularly at elevated GFAP. Lower LC NE transporter‐weighted connectivity with the cortex was associated with faster cognitive decline in individuals with elevated GFAP and ptau217 (Figure 2).

**Conclusion:**

These findings suggest that LC‐NE dysfunction contributes to increases in tau and its downstream effects on cognition, facilitated by astrocyte reactivity starting from the earliest stages of the disease. Maintaining optimal LC functioning may hold promise to delay disease progression.